# MHANet: A hybrid attention mechanism for retinal diseases classification

**DOI:** 10.1371/journal.pone.0261285

**Published:** 2021-12-16

**Authors:** Lianghui Xu, Liejun Wang, Shuli Cheng, Yongming Li

**Affiliations:** College of Information Science and Engineering, Xinjiang University, Urumqi, China; Newcastle University, UNITED KINGDOM

## Abstract

With the increase of patients with retinopathy, retinopathy recognition has become a research hotspot. In this article, we describe the etiology and symptoms of three kinds of retinal diseases, including drusen(DRUSEN), choroidal neovascularization(CNV) and diabetic macular edema(DME). In addition, we also propose a hybrid attention mechanism to classify and recognize different types of retinopathy images. In particular, the hybrid attention mechanism proposed in this paper includes parallel spatial attention mechanism and channel attention mechanism. It can extract the key features in the channel dimension and spatial dimension of retinopathy images, and reduce the negative impact of background information on classification results. The experimental results show that the hybrid attention mechanism proposed in this paper can better assist the network to focus on extracting thr fetures of the retinopathy area and enhance the adaptability to the differences of different data sets. Finally, the hybrid attention mechanism achieved 96.5% and 99.76% classification accuracy on two public OCT data sets of retinopathy, respectively.

## Introduction

Diabetic retinopathy (DR) is a common blinding disease, mostly in diabetic patients [1]. Changes in blood components of diabetic patients cause dysfunction of vascular endothelial cells, which leads to impaired retinal barrier in diabetic patients. According to a study by the International Diabetes Federation (IDF), patients with diabetes over 10 years have a 60.00% chance of developing retinopathy. Periodic examinations of diabetic patients and effective control of retinal diseases can prevent patients from transient or permanent blindness [2]. Diabetic macular edema (DME) is one of the main symptoms of diabetic retinopathy (DR) [3]. The main reason for this disease is the long-term hyperglycemia state of diabetic patients, which leads to increased vascular permeability of the retina and choroid of the eye, and the water molecules and some protein components in the blood can permeate through the damaged vascular walls and form edema in the macular area.

Another common retinopathy is age-related macular degeneration (AMD) [4, 5]. At present, the exact cause of the disease is unknown. It may be related to genetic factors, environmental effects, chronic retinal light injury, nutritional disorders, metabolic disorders and so on. However, aging and degeneration are important factors that cause age-related macular degeneration. According to clinical manifestations and pathological changes, drusen(DRUSEN) and choroidal neovascularization (CNV) are one of the main symptoms of AMD.

Among them, the early symptoms of CNV are subretinal neovascularization, but the symptoms are not obvious. In the middle stage, the blood vessels will gradually expand to a certain extent, causing leakage, rupture and bleeding, which can lead to vision loss. In the later stage, with the aggravation of vascular infiltration and bleeding, the patient’s vision was severely damaged, which caused the patient’s permanent visual impairment.

DRUSEN is often caused by metabolic disorders of the pigment epithelium. In the early stage, the patient’s visual function may not be impaired. However, as the condition worsens, some patients have enlarged physiological blind spots in their eyes. If the situation is more serious, it may cause the patient’s field of view to narrow or vision loss. AMD has become the most important blinding disease in western developed countries [4, 5]. As the macular area is the most important part of the retina and the most sensitive part of vision, 80.00% of human vision comes from the macular area. Once a lesion such as edema occurs in the macular area, the impact on vision is very serious. Therefore, regular checking for diabetic patients and effective control of the disease in early stage can prevent temporary or permanent blindness in [2].

With the rapid increase in the number of patients with retinopathy, hospitals need a lot of manpower and material resources to face complex situations. In order to reduce the burden on hospitals and doctors, the auxiliary diagnosis and analysis technology of retinopathy is an urgent technology for doctors. OCT is a new tomography technology developed in recent years [6–9]. Compared with the traditional fundus detection technology, OCT can perform non-contact and non-invasive tomography on the microstructure of living eye tissue [10]. Therefore, this paper uses the OCT retinal fundus detection image datasets for our research.

With the wide application of neural networks in many fields [11], medical image classification based on deep learning has also become a research hotspot. Many scholars have tried to use neural networks to identify retinopathy, and achieved good results. In order to better lock foreground information of the picture, image denoisingis often used in image processing [12, 13]. In addition, the attention mechanism can also lock foreground information of the image very well, so this article explores the role of the attention mechanism in the recognition of retinal diseases. Attention mechanism was originally used in machine translation [14], and now it has become an important concept in image recognition. When recognizing an image, it is similar to the human visual system. It can assist the network to pay more attention to the key information in the image. In order to better identify retinal diseases, this paper proposes a new attention mechanism, which can better improve the accuracy of retinal disease recognition.

At present, the attention mechanism in image recognition is mainly divided into channel attention mechanism and spatial attention mechanism. The channel attention mechanism is used to distinguish the relationship between the feature map channels and the spatial attention mechanism is used to distinguish the relationship between the elements in the feature map. Because the lesion area in OCT images of retinopathy has the following characteristics: (1) The lesion features are concentrated in the local area. (2) The characteristics of the lesions are not much different from those of normal tissues. Therefore, this paper proposes a multi-branch hybrid attention network (MHANet) to lock the lesion area of the picture. Specifically, the channel attention mechanism is mainly used to highlight the channel elements with the most abundant lesion features; Spatial attention mechanism is mainly used to highlight the elements of the area where the lesion features are located. Experimental results show that this algorithm has certain advantages compared with previous mainstream algorithms. In addition, the visualization results show that compared with the mainstream model using channel attention mechanism alone, the hybrid attention mechanism proposed in this paper can more accurately lock the location of the lesion area in retinal OCT images.

## Related work

With the development of machine learning and deep learning, medical image analysis based on deep learning has become a research hotspot for many scholars.

In 2014, Srinivasan et al. [15] applied machine learning methods to image analysis of retinal diseases. They used the support vector machine (SVM) as the classification tool and the HOG feature of the OCT images as the classification basis to classify and recognize the three types of retinopathy images. They constructed a multi class classifier using three linear SVM, and then classified NORMAL and AMD, NORMAL and DME, AMD and DME. Finally, AMD, DME and NORMAL achieved 100.00%, 100.00% and 86.67% classification accuracy respectively. This experimental method not only increases the complexity of the experiment but also does not fundamentally distinguish the three types of pictures at the same time. In addition, in order to promote research of ophthalmic diseases, they also created a public OCT dataset for the majority of scholars to use.

In 2016, Wang et al. [16] used same data set as Srinivasan et al. [15] to further deepen research on retinopathy. They first used two feature selection algorithms CFS (Correlation-based Feature Subset) and CSE (Classifier Subset Evaluator) to find a subset of Linear Configuration Pattern (LCP) features [17, 18], and then used the Sequence Minimization Optimization (SMO) algorithm as a classification tool to recognize and classify the selected image features. Unlike Srinivasan et al., they combine the local and global features of the picture. The experimental results show that Wang et al achieved 97.80%, 94.00% and 99.60% accuracy on AMD, DME and NORMAL respectively. Although the feature selection algorithm can reduce the redundant or useless features in the picture, some key features will be lost in the process of feature selection.

In 2017, Rasti et al. [13] used a multi-scale neural network to analyze retinopathy images. They used parallel convolution kernels of different sizes to extract the features of different regions of the feature map, and then set up a gated network to assign corresponding coefficients to the feature maps extracted by different convolution kernels to adjust the relationship between different feature maps. In addition, they also created a new OCT retinal disease dataset. For the dataset created by Srinivasan et al., the overall classification accuracy of AMD, DME and NORMAL is 99.39%. For their own datasets, the accuracy rates of AMD, DME, and NORMAL are 95.67%, 98.22% and 96.67%, respectively. Although the gated network can reconcile the relationship between the feature map and the feature map, it does not highlight the importance of the channel or region of the feature map itself.

In 2017, Karri et al. [19] used a fine-tuning pre-training model (GoogleNet) to study the OCT images of retinopathy provided by Srinivasan et al. They first changed the fully connection layer of the last layer of the network to 3 outputs, then called the parameters pre-trained on ImageNet in advance as the initialization parameters of the network, and finally used the retinopathy dataset for network training. The accuracy of the final experiment on AMD, DME and NORMAL were 89.00%, 86.00% and 99.00% respectively. Although the pre training model can make the network quickly reach the convergence state, the final classification effect needs to be improved.

In 2019, Feng et al. [20] conducted a classification and recognition study on four types of eye retina images. They first loaded the pre training parameters of VGG16 trained in Imagenet, and then changed the full connection layer of the last layer to four outputs. Finally, the overall accuracy of 97.80% was achieved on CNV, DME, DRUSEN and NORMAL. Although the migration learning method proposed in literature [19, 20] can reduce the training time of the network and alleviate the network’s excessive dependence on the dataset, it reduces the generalization of the network to different datasets. For OCT image classification research, Wang et al. [21] compared VGG16 [22], VGG19 [22], Inception-v3 [23] with CliqueNet [24], DPN92 [25], DenseNet121 [26], ResNet50 [27], ResNext101 [28]. Experiments show that the network with jump connection operation can reduce the loss of effective information in the process of feature extraction and significantly improve the classification effect.

With the development of a deep learning network, current scholars are pursuing the lightness and convenience of the network. Ding X et al. [29] proposed the RepVGG network. RepVGG adds a jump connection operation to the network based on the VGG network. On the premise of ensuring training speed and training accuracy, the accuracy of the RepVGG network on ImageNet dataset can reach more than 80%, therefore, this article uses the RepVGG network to study the retina OCT images.

In addition, the outstanding achievements of attention mechanism in the field of image have also attracted the interest of many scholars. In 2019, Jie et al. [30] proposed SENet network architecture. The core idea of the network is to assign the corresponding channel coefficients to the multi-channel feature maps, so as to highlight the relationship between the channels of the feature maps. Soon after the SENet network was proposed, its enhanced network architecture SKNet [31] was born. Compared with SEnet, SKNet studies the channel-to-channel relationship between two feature maps and has achieved better results on the ImageNet dataset.

According to the characteristics of lesion areas in OCT retinopathy images, we propose a hybrid attention mechanism, which is composed of a channel attention mechanism and a spatial attention mechanism in parallel. It mainly has the following characteristics:

1. By calculating the channel coefficients of the feature maps extracted by the convolution kernels with different expansion rates, the network can automatically identify the importance of the corresponding channel elements between different feature maps.
2. By calculating the spatial coefficients of the feature maps extracted by the convolution kernels of different sizes, the network can automatically identify the importance of regional elements in different feature maps.

## Material and methods

### Datasets

Considering the influence of the number of datasets on the experiment, two public datasets are used in this paper. Among them, Dataset1 was provided by Rasti et al., obtained at the Noor Eye Hospital in Tehran using Spectralis SD-OCT imaging [13]. Dataset2 was provided by Kermany et al, and obtained by using Spectralis SD-OCT imaging [32].

The original train dataset of Dataset2 is 83484 OCT images from 4686 patients. There are 37,205 choroidal neovascularization (CNV), 8616 drusen (DRUSEN), 11348 diabetic macular edema (DME), and 26315 normal(NORMAL). The test dataset includes 250 pictures of CNV, DRUSEN, DME and NORMAL from 633 patients. In the training stage, due to the huge difference in the number of four types of pictures, the model focuses on learning large sample data, so that small sample data is not fully learned, and generalization ability of the network is reduced. Therefore, we processed Dataset2 as follows: 1: Using 8616 DRUSEN images as the standard, 8616 images were randomly selected from CNV, DME and NORMAL. 2: The number of each picture is 8616, we divide it into train dataset and test dataset according to the ratio of 8:2.

Dataset1 has a total of 4254 images, of which 1104 are DME, 1565 are AMD, and 1585 are NORMAL. Similarly, we divide the pictures into train dataset and test dataset according to the ratio of 8:2. Table 1 shows the main statistics of the two datasets in this article.

**Table 1:** Dataset statistics.

Dataset		AMD	DME	NORMAL	
Dataset1	Train	1252	884	1268	
	Test	313	220	317	
		CNV	DRUDEN	DME	NORMAL
Dataset2	Train	6893	6893	6893	6893
	Test	1723	1723	1723	1723

**Fig 1:**
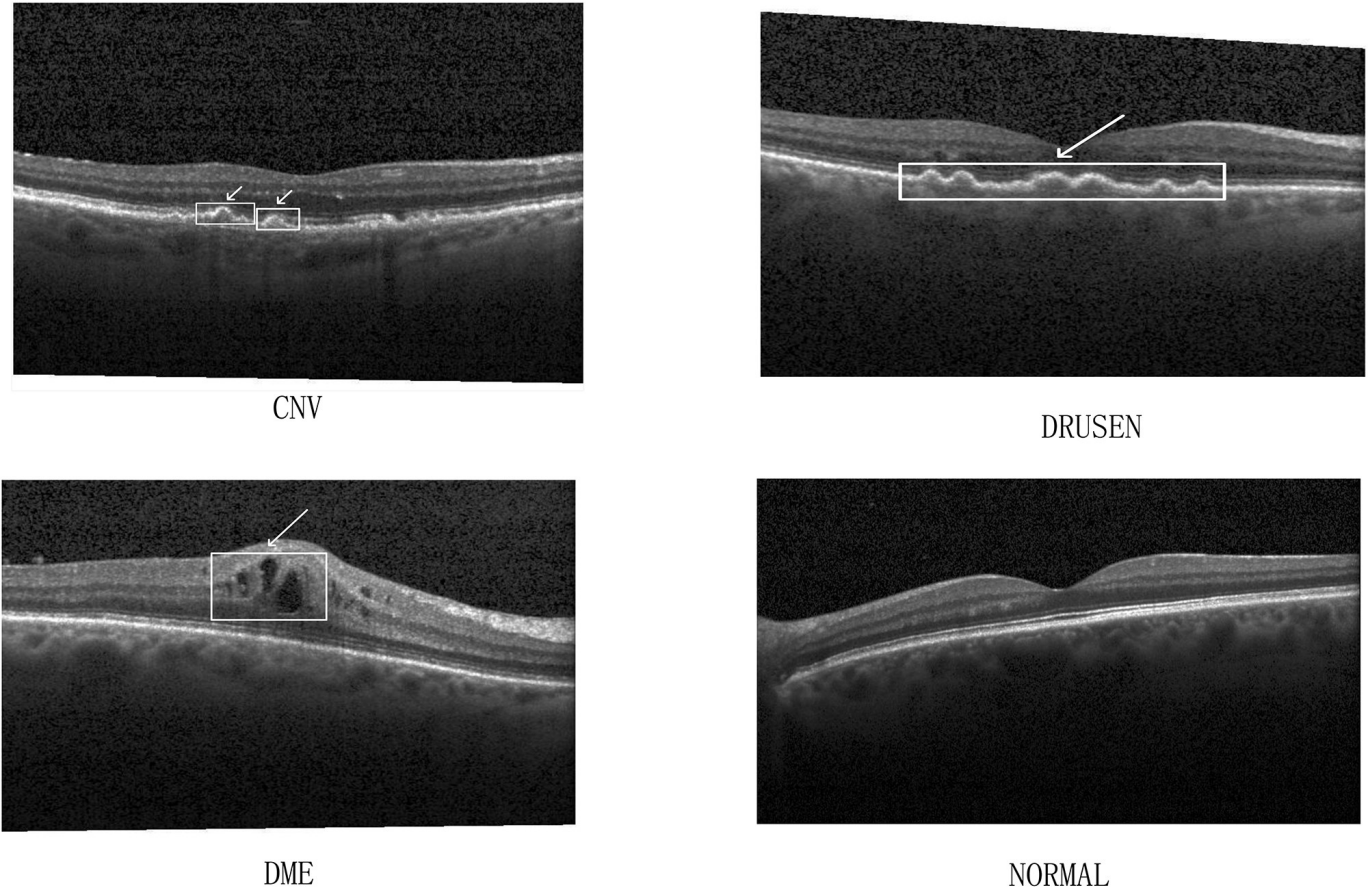
Sample demo of the dataset.

Since the quality and size of the two public data sets are different, we used these two data sets for training and evaluation respectively. Experimental data shows that our network model has good generalization ability. Fig 1 show examples of datasets.

### Network architecture

**Fig 2:**
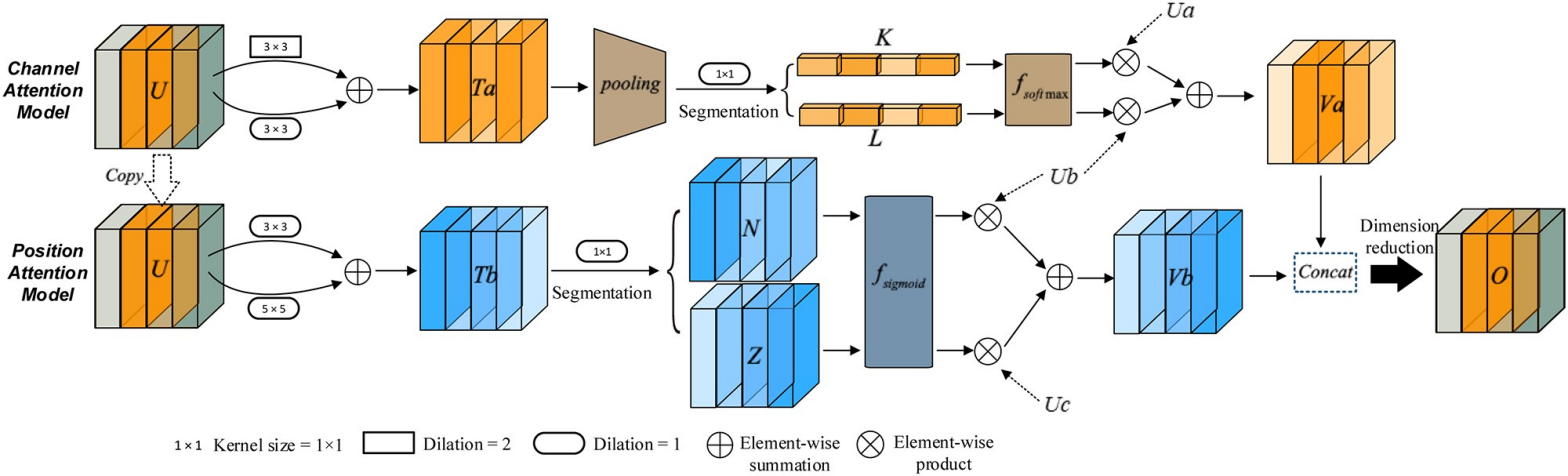
Overview of the proposed structure Multi-branch hybrid attention network.

In this section, we will introduce our proposed Multi-branch hybrid attention network (MHANet) in detail. As shown in Fig 2, MHANet consists of two parts: channel attention module and spatial attention module. The main function of the channel attention module is to assign channel coefficients to different feature maps to identify the channel-to-channel correlation between the feature maps. The main function of the spatial attention module is to assign spatial coefficients to different feature maps to distinguish the importance of location information between the feature maps.

As shown in Fig 2, *U* ∈ *R*^*C*×*H*×*W*^ is the feature map, C is the number of channels,W and H represent length and width of the feature map, respectively. First, we performed three convolution operations on the input feature map: *F*1: *U* → *Ua* ∈ *R*^*C*×*H*×*W*^, *F*2: *U* → *Ub* ∈ *R*^*C*×*H*×*W*^, and *F*3: *U* → *Uc* ∈ *R*^*C*×*H*×*W*^. Among them, F1 means using a convolution operation with a convolution kernel size of 3 × 3 and an expansion rate of 2. F2 represents a convolution operation with a convolution kernel size of 3 × 3 and an expansion rate of 1. F3 represents a convolution operation with a convolution kernel size of 5 × 5 and an expansion rate of 1. Secondly, the element summation operation is carried out, and the feature maps from different branches are fused to obtain *Ta* ∈ *R*^*C*×*H*×*W*^ and *Tb* ∈ *R*^*C*×*H*×*W*^ respectively.

#### Channel attention model of MHANet

In order to identify the channel-to-channel relationship between different feature maps, we use feature maps *Ta* ∈ *R*^*C*×*H*×*W*^ to obtain channel attention coefficients. The specific operations are shown in the following steps:

In order to reduce redundant information in the feature map and improve fault tolerance of the model, we first perform the maximum pooling and average pooling operations on the feature map *Ta* in the channel dimension to obtain two new feature maps {A, E} ∈ *R*^C×1×1^. Then we add and fuse the two new feature maps so that we can get foreground information of the picture as much as possible.

$$\begin{array}{c} A=\text{Max}(Ta) \end{array}$$ (1)

$$\begin{array}{c} E=\text{Mean}(Ta) \end{array}$$ (2)

$$\begin{array}{c} Pa_{i}=A_{i}+E_{i},i\in \left\{0,\left(C-1\right)\right\} \end{array}$$ (3)

$$\begin{array}{c} Pa=[Pa_{0},Pa_{1},\ldots ,Pa_{C-1}],Pa_{i}\in R^{1\times 1} \end{array}$$ (4)

The maximum pooling and average pooling in the channel dimension are to take a maximum value and an average value from the feature map element values of each channel respectively; *A*_*i*_ ∈ *R*^1×1^ and *E*_*i*_ ∈ *R*^1×1^ are obtained from the i-th channel feature map of *Ta* through maximum pooling and average pooling respectively.

In addition, in order to assign channel coefficients to the feature maps *Ua* and *Ub*, we perform a convolution operation on the feature map *Pa* to achieve the channel dimension upgrade to obtain the feature map *Pb* ∈ *R*^2*C*×1×1^, in which the size of the convolution kernel is 1 × 1. Then the feature map *Pb* is divided equally in the channel dimension to obtain the feature maps *K* ∈ *R*^*C*×1×1^ and *L* ∈ *R*^*C*×1×1^.

$$\begin{array}{c} K_{i}=Pb_{i},i\in \left\{0,\left(C-1\right)\right\} \end{array}$$ (5)

$$\begin{array}{c} L_{i}=Pb_{i+C} \end{array}$$ (6)

$$\begin{array}{c} Pb=[Pb_{0},Pb_{1},\ldots ,Pb_{C-1},Pb_{C},Pb_{C+1},\ldots ,Pb_{2C-1}] \end{array}$$ (7)

Among them, *Pb*_*i*_ ∈ *R*^1×1^ and *Pb*_*i*+*C*_ ∈ *R*^1×1^ represent the element values in the *i* and *i* + *C* channels of the feature map *Pb* respectively; *K*_*i*_ ∈ *R*^1×1^ and *L*_*i*_ ∈ *R*^1×1^ represent the element values in the i-th channel of the feature maps K and L respectively.

Finally, we perform a Softmax operation on K and L to obtain the channel attention coefficients *A* tta ∈ *R*^*C*×1×1^ and *A* ttb ∈*R*^*C*×1×1^, and then we multiply *Atta* and *Attb* with *Ua* and *Ub* respectively, and the two feature maps obtained after the multiplication are added and fused to obtain the feature map *Va* ∈ *R*^*C*×*H*×*W*^.

$$\begin{array}{c} A\text{tt}a_{i}=\frac{e^{K_{i}}}{e^{K_{i}}+e^{L_{i}}},A{\text{ttb}}_{i}=\frac{e^{L_{i}}}{e^{K_{i}}+e^{K_{i}}} \end{array}$$ (8)

$$\begin{array}{c} Va_{i}=Atta_{i}\times Ua_{i}+Attb_{i}\times Ub_{i} \end{array}$$ (9)

$$\begin{array}{c} Va=[Va_{0},Va_{1},\ldots ,Va_{C-1}],\;Va_{i}\in R^{H\times W} \end{array}$$ (10)

Atta_*i*_ ∈ *R*^1×1^ and Att *b*_*i*_ ∈ *R*^1×1^ respectively represent the corresponding coefficients obtained from the element values *K*_*i*_ and *L*_*i*_, and Atta_*i*_ + *Attb*_*i*_ = 1; *Ua*_*i*_ ∈ *R*^*H*×*W*^ and *Ub*_*i*_ ∈ *R*^*H*×*W*^ respectively represent all the element values of the i-th channel of the feature maps *Ua* and *Ub*.

#### Position attention model of MHANet

In order to enable the network to distinguish the importance of elements in different feature maps, we use the feature map *Tb* ∈ *R*^*C*×*H*×*W*^ to calculate the spatial attention coefficient. The specific operation includes the following steps.

In order to assign spatial coefficients to the feature maps *Ua* and *Ub*, we perform a convolution operation on the feature map *Tb* to achieve the channel dimension upgrade to obtain the feature map *M* ∈ *R*^2*C*×*H*×*W*^, in which the size of the convolution kernel is 1 × 1. Then the feature map M is divided equally in the channel dimension to obtain the feature maps *N* ∈ *R*^*C*×*H*×*W*^ and *Z* ∈ *R*^*C*×*H*×*W*^.

$$\begin{array}{c} N_{j}=M_{j},j\in \left\{0,\left(C-1\right)\right\} \end{array}$$ (11)

$$\begin{array}{c} Z_{j}=M_{j+C} \end{array}$$ (12)

$$\begin{array}{c} M\in [M_{0},M_{1},\ldots ,M_{C-1},M_{C},M_{C+1},\ldots ,M_{2C-1}\} \end{array}$$ (13)

*M*_*j*_ ∈ *R*^*H*×*W*^ and *M*_*j*+*C*_ ∈ *R*^*H*×*W*^ respectively represent the *H* × *W* element values in the *j* and *j* + *C* channels of the feature map *M*. *N*_*j*_ ∈ *R*^*H*×*W*^ and *Z*_*j*_ ∈ *R*^*H*×*W*^ represent all the element values in the j-th channel of the feature maps *N* and *Z* respectively.

Finally, we use the Sigmoid activation function on the feature maps *N* and *Z* to obtain the spatial attention coefficients *Attc* ∈ *R*^*C*×*H*×*W*^ and *Attd* ∈ *R*^*C*×*H*×*W*^, and then multiply the spatial attention coefficients *Attc* and *Attd* with the feature maps *Ub* and *Uc* respectively, and the two feature maps obtained after the multiplication are added and fused to obtain the feature map *Vb* ∈ *R*^*C*×*H*×*W*^.

$$\begin{array}{c} {\text{Attc}}_{\left(j,h,w\right)}=\frac{1}{1+e^{N_{\left(j,h,w\right)}}},j\in \left\{0,\left(C-1\right)\right\},h\in \left\{0,\left(H-1\right)\right\},w\in \left\{0,\left(W-1\right)\right\} \end{array}$$ (14)

$$\begin{array}{c} {\text{Attd}}_{\left(j,h,w\right)}=\frac{1}{1+e^{Z_{\left(j,h,w\right)}}} \end{array}$$ (15)

$$\begin{array}{c} Vb_{j}=Attc_{j}\times Ub_{j}+Attd_{j}\times Uc_{j} \end{array}$$ (16)

$$\begin{array}{c} Vb=[Vb_{0},Vb_{1},\ldots ,Vb_{C-1}],Vb_{j}\in R^{H\times W} \end{array}$$ (17)

*N*_(*j*,*h*,*w*)_ and *Z*_(*j*,*h*,*w*)_ respectively represent the element at the position (*h*, *w*)∘ in the j-th channel of the feature maps N and Z; *Attc*_(*j*,*h*,*w*)_ and Attd _(*j*,*h*,*w*)_ are obtained from the corresponding *N*_(*j*,*h*,*w*)_ and *Z*_(*j*,*h*,*w*)_ respectively. The specific formula is shown above.

Finally, we concatenate the feature maps *V*1 and *V*2 to obtain the feature map *V* ∈ *R*^2*C*×*H*×*W*^, and then use the convolution operation with the convolution kernel size of 1 × 1 to reduce the channel dimension of the feature map *V* to obtain the feature map *O* ∈ *R*^*C*×*H*×*W*^. It is worth noting that the final output feature map highlights the foreground information of the picture in both the channel dimension and the spatial dimension.

In addition, we provide pseudo code to introduce the overall flow of the experiment, as shown in Table 2.

**Table 2:** Training algortithm.

**Tlgorithm 1** Training algortithm of **MHANet**
1:**for** number of training epochs **do** 2: **for** k steps **do** 3: Sample a batch of images **X**_1_,**X**_2_,…,**X**_*n*_ and corresponding labels $Y_{1}^{true}$,$Y_{2}^{true}$,…,$Y_{n}^{true}$ from the training dataset. 4: **X**_1_,**X**_2_,…,**X**_*n*_ are resized into a size of 224*224 and $X_{1}^{normal}$,$X_{2}^{normal}$,…,$X_{n}^{normal}$ are obtained with the same size. 5: Input $X_{1}^{normal}$,$X_{2}^{normal}$,…,$X_{n}^{normal}$ into the hybrid attention mechanism network **MHANet**, and obtain output value $Y_{1}^{predict}$,$Y_{2}^{predict}$,…,$Y_{n}^{predict}$. 6: Use the cross-entropy loss function to calculated true lable $Y_{1}^{true}$,$Y_{2}^{true}$,…,$Y_{n}^{true}$ and the predicted label $Y_{1}^{predict}$,$Y_{2}^{predict}$,…,$Y_{n}^{predict}$, and then use the SGD gradient descent method to update the network parameters. 7: end for 8: end for

## Experiment and statistical analysis method

### Experimental details

The experiments were run based on the Python 3.6, Torch 1.6.0. In the process of network training, after many experiments, it is found that the learning rate is 0.001, the batch size is set to 224, and the network can achieve the optimal effect. The loss function is ‘cross-entropy loss’, and the optimization method is the SGD gradient descent method.

### Statistical analysis method

Because different evaluation indexes have different evaluation significance to the experimental results, three common evaluation indexes are used to evaluate the experimental results in this paper: accuracy rate, precision rate, and recall rate. Also, to make the experiment more concise and understandable, we use the mixed matrix to show the classification results of each kind of data. Accuracy, precision, and recall are defined as follows:

$$\begin{array}{c} \;\text{Accuracy}\;=\frac{TP+TN}{TP+TN+FP+FN} \end{array}$$ (18)

$$\begin{array}{c} \text{Precision}=\frac{TP}{TP+FP} \end{array}$$ (19)

$$\begin{array}{c} \text{Rec}\;all=\frac{TP}{TP+FN} \end{array}$$ (20)

$$\begin{array}{c} F1=2\frac{\;\text{Pre}\;\text{cision}\;\times \text{Rec}\;\text{all}\;}{\;\text{Pre}\;\text{cision}\;+\text{Rec}\;\text{all}\;} \end{array}$$ (21)

TP represents the number of samples that are actually positive and predicted to be positive; TN represents the number of samples that are actually negative and predicted to be negative; FP represents the number of samples that are actually negative and predicted to be positive; FN represents the number of samples that are actually positive and predicted to be negative.

## Experimental results

In order to better illustrate the positive effect of the hybrid attention mechanism on the recognition of retinopathy, we give the classification accuracy of the entire dataset and the classification index score of each type of data.

From the accuracy score of each model on Dataset1, it can be seen that the attention mechanism plays an active role in the three types of image recognition of AMD, DME, and NORMAL. In particular, our proposed MHANet network achieved the best results in retinal image classification, and the overall accuracy reached 99.76%, as shown in Table 3.

**Table 3:** Accuracy of Dataset1.

Dataset	Model	ACC(%)
Dataset1	VGG16	96.47
	RepVGG	90.82
	ResNet50	98.00
	Res2Net50	97.05
	SENet	97.41
	SKNet	99.05
	MHANet(our)	**99.76**

Table 4 shows the Precision, Recall, and F1 of each type of picture. It can be seen from the table that the F1 values obtained by ResNet50, Res2Net50, SENet and SKNet on AMD and NORMAL are significantly better than their F1 values obtained on DME, which indicates that generalization ability of the above models has certain defects, and hybrid attention proposed in this article effectively alleviates this problem.

**Table 4:** Precision, Recall, F1 of Dataset1.

Dataset	Model	Precision(%)	Recall(%)	F1((%)
AMD	VGG16	97.74	97.12	97.43
	RepVGG	91.16	92.33	91.74
	ResNet50	99.35	97.76	98.55
	Res2Net50	98.37	96.80	97.58
	SENet	99.01	96.48	97.73
	SKNet	99.67	98.72	99.19
	MHANet(our)	**1.00**	**99.36**	**99.67**
DME	VGG16	95.92	96.36	96.14
	RepVGG	93.17	86.81	89.88
	ResNet50	96.00	98.18	97.07
	Res2Net50	94.32	98.18	96.21
	SENet	94.71	97.72	96.19
	SKNet	96.90	99.54	98.20
	MHANet(our)	**99.09**	**1.00**	**99.54**
NORMAL	VGG16	95.59	95.89	95.74
	RepVGG	89.00	92.11	90.54
	ResNet50	98.10	98.106	98.10
	Res2Net50	97.76	96.52	97.14
	SENet	97.79	98.10	97.95
	SKNet	**1.00**	99.05	99.52
	MHANet(our)	**1.00**	**1.00**	**1.00**

For the Dataset2, the four types of pictures are divided according to the ratio of 1:1:1:1, so the network will not have the over fitting problem caused by the large difference in the number of training pictures. The MHANet proposed in this paper adopts both spatial attention mechanism and channel domain attention mechanism, and achieves the best results. See Table 5 for specific experimental data.

**Table 5:** Accuracy of Dataset2.

Dataset	Model	ACC(%)
Dataset1	VGG16	92.19
	RepVGG	94.45
	ResNet50	95.31
	Res2Net50	95.47
	SENet	95.24
	SKNet	95.40
	MHANet(our)	**96.51**

**Fig 3:**
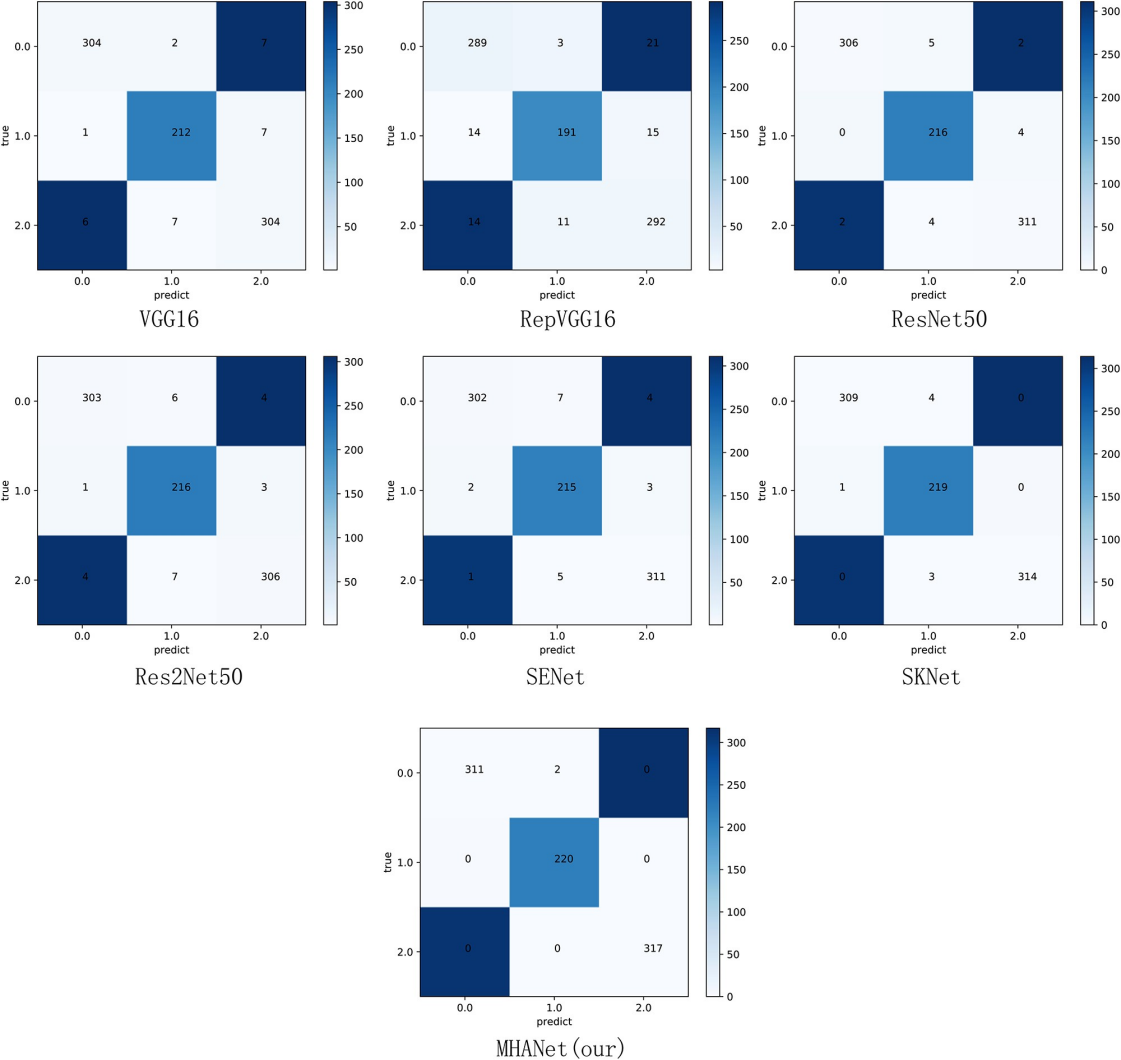
Confusion matrix of the Dataset1.

**Fig 4:**
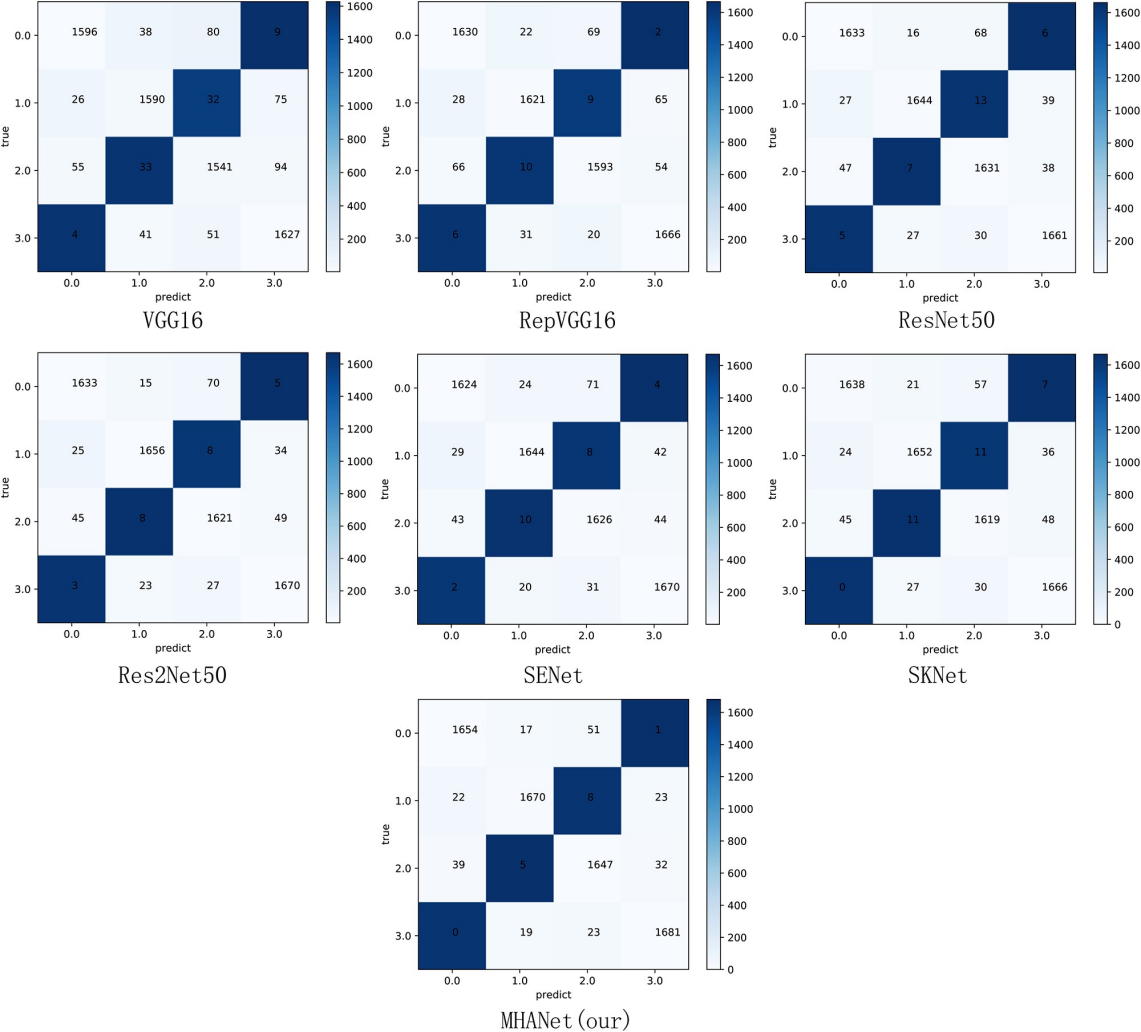
Confusion matrix of the Dataset2.

As shown in Table 6, the MHANet network proposed in this paper not only achieved the best results in accuracy, but also has certain improvements in Precision, Recall, and F1 compared to other networks. This is because the MHANet network can not only automatically highlight the importance of channels, but also distinguish the importance of element values in the entire feature map. In order to better prove the feasibility of the innovation in this paper, this paper further demonstrates the experimental results of Dataset1 and Dataset2 through a confusion matrix. The results are shown in Figs 3 and 4.

**Table 6:** Precision, Recall, F1 of Dataset2.

Dataset	Model	Precision(%)	Recall(%)	F1((%)
CNV	VGG16	94.94	92.62	93.77
	RepVGG	94.21	94.60	94.41
	ResNet50	95.38	94.77	95.08
	Res2Net50	95.72	94.77	95.24
	SENet	95.64	94.25	94.94
	SKNet	95.95	95.06	95.51
	MHANet(our)	**96.44**	**95.99**	**96.21**
DRUSEN	VGG16	93.41	92.28	92.84
	RepVGG	96.25	94.08	95.15
	ResNet50	97.04	95.41	96.22
	Res2Net50	97.29	96.11	96.70
	SENet	96.81	95.41	96.11
	SKNet	96.55	95.87	96.21
	MHANet(our)	**97.60**	**96.92**	**97.26**
DME	VGG16	90.43	89.43	89.93
	RepVGG	94.20	92.45	93.32
	ResNet50	93.62	94.66	94.14
	Res2Net50	93.91	94.08	93.99
	SENet	93.66	94.37	94.01
	SKNet	94.29	93.96	94.12
	MHANet(our)	**95.25**	**95.58**	**95.42**
NORMAL	VGG16	90.13	94.42	92.23
	RepVGG	93.22	96.69	94.92
	ResNet50	95.24	96.40	95.81
	Res2Net50	94.99	96.92	95.94
	SENet	94.88	96.92	95.89
	SKNet	94.82	96.69	95.74
	MHANet(our)	**96.77**	**97.56**	**97.16**

As shown in Fig 3, we give the confusion matrix obtained by each model based on Dataset1, where 0.0 represents AMD, 1.0 represents DME and 2.0 represents NORMAL. From each confusion matrix, we can know that other models will more or less misclassify these three types of pictures. MHANet proposed in this paper can completely and correctly classify DME and NORMAL, and minimize the error of AMD as DME.

As shown in Fig 4, we give the confusion matrix obtained by each model based on Dataset2. Among them, 0.0 represents CNV, 1.0 represents DRUSEN, 2.0 represents DME, and 3.0 represents NORMAL. From the perspective of the model, the MHANet model has the largest number of correct image classifications among the five models. Among them, MHANet has 298 correctly classified pictures more than VGG16, 142 correctly classified pictures more than RepVGG, 80 correctly classified pictures more than ResNet50, 72 correctly classified pictures more than Res2Net50, 88 correctly classified pictures more than SENet and 77 correctly classified pictures more than SKNet. This fully proves that this model has achieved the best effect in the task of retinal disease recognition. In addition, other models are prone to mispredict sample 2 as sample 0 and mispredict sample 3 as sample 2. For these two kinds of images that are easy to distinguish errors, MHANet has the best classification effect.

**Fig 5:**
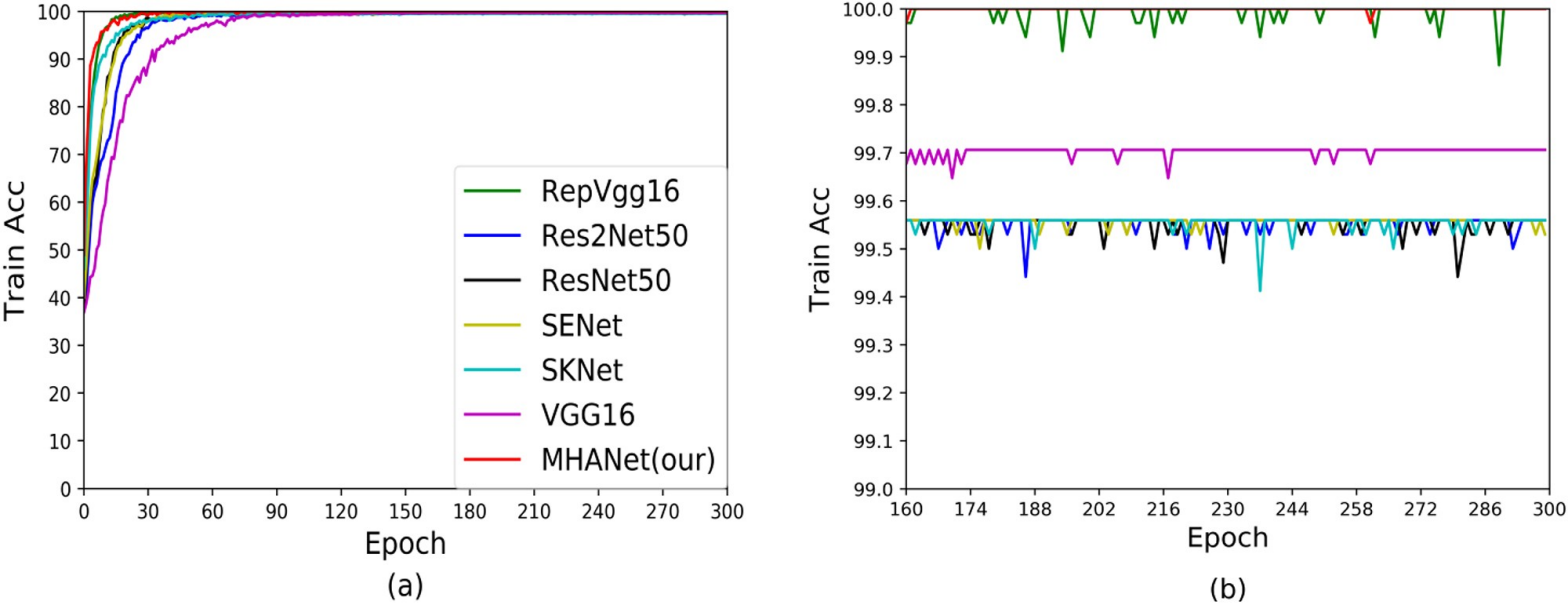
Training accuracy curves of Dataset1.

**Fig 6:**
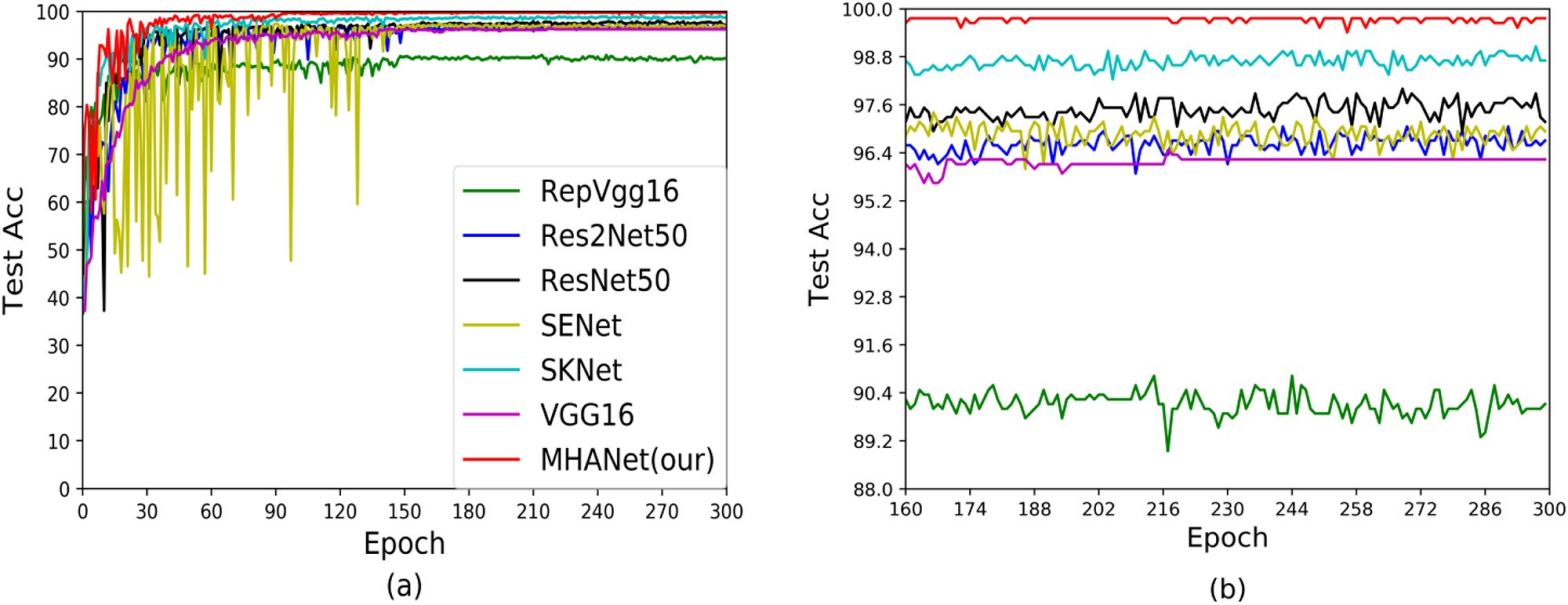
Test accuracy curves of Dataset1.

**Fig 7:**
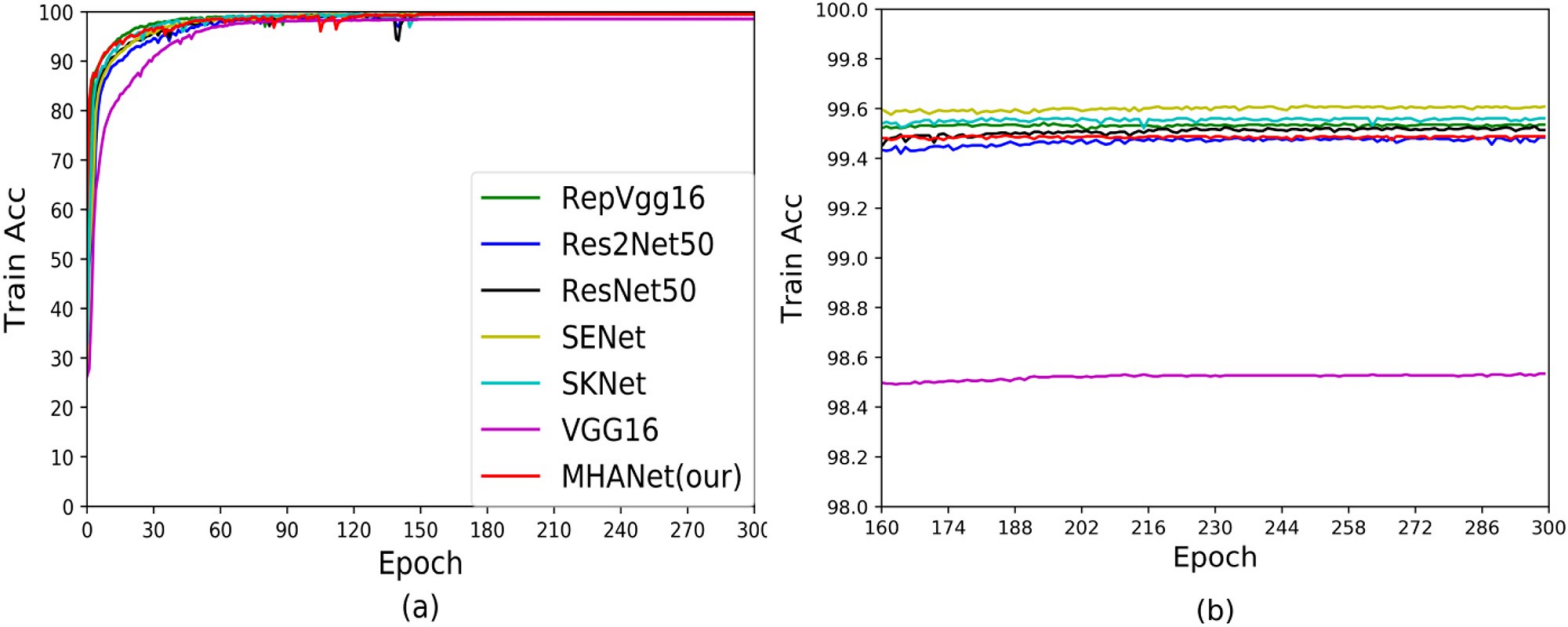
Training accuracy curves of Dataset2.

**Fig 8:**
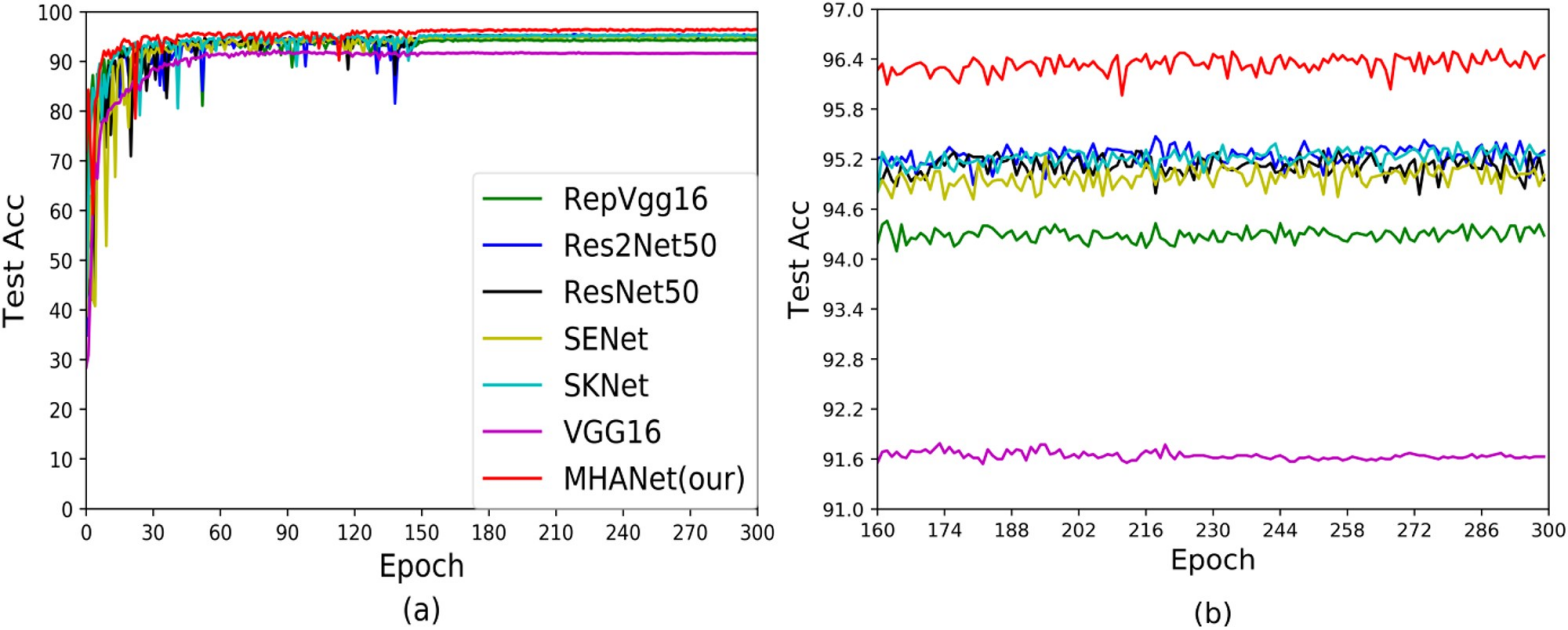
Test accuracy curves of Dataset2.

In order to analyze the generalization ability of the model, we give the accuracy curve during model training and the accuracy curve during testing, as shown in Figs 5–8. Among them, Figs 5 and 6 are the accuracy curves during training and testing based on Dataset1. It can be seen from the figure that VGG16 and RepVGG can get very high scores during training, but the test results are not satisfactory, indicating that generalization ability of VGG16 and RepVGG on Dataset1 is not strong. ResNet50 and Res2Net50 have achieved good results in both the train stage and the test stage, indicating that ResNet50 and Res2Net50 have better generalization capabilities on Dataset1, but ResNet50 is better than Res2nNet50 on the test set. This is because Res2Net50 has more parameters than ResNet50, which makes it difficult for the network to reach the best state. Similarly, it can be seen from the figure that SENet and SKNet have good generalization ability on Dataset1, but the test results have not been greatly improved, so the network has certain limitations. Finally, we can see that the MHANet network can get the best results in the train stage and the test stage, which proved that MHANet has good convergence and has made breakthrough.

Figs 7 and 8 show the accuracy during training and testing based on Dataset2. It can be seen from the figure that compared with Dataset1, RepVGG performed better on Dataset2. In addition, ResNet50, Res2Net50, SENet, SKNet and MHANet achieved convergence in the train and test stages, but MHANet achieved the best experimental results in the testing phase. This once again proved that the MHANet network can get good experimental results on small datasets, and good results can be obtained on large datasets.

**Fig 9:**
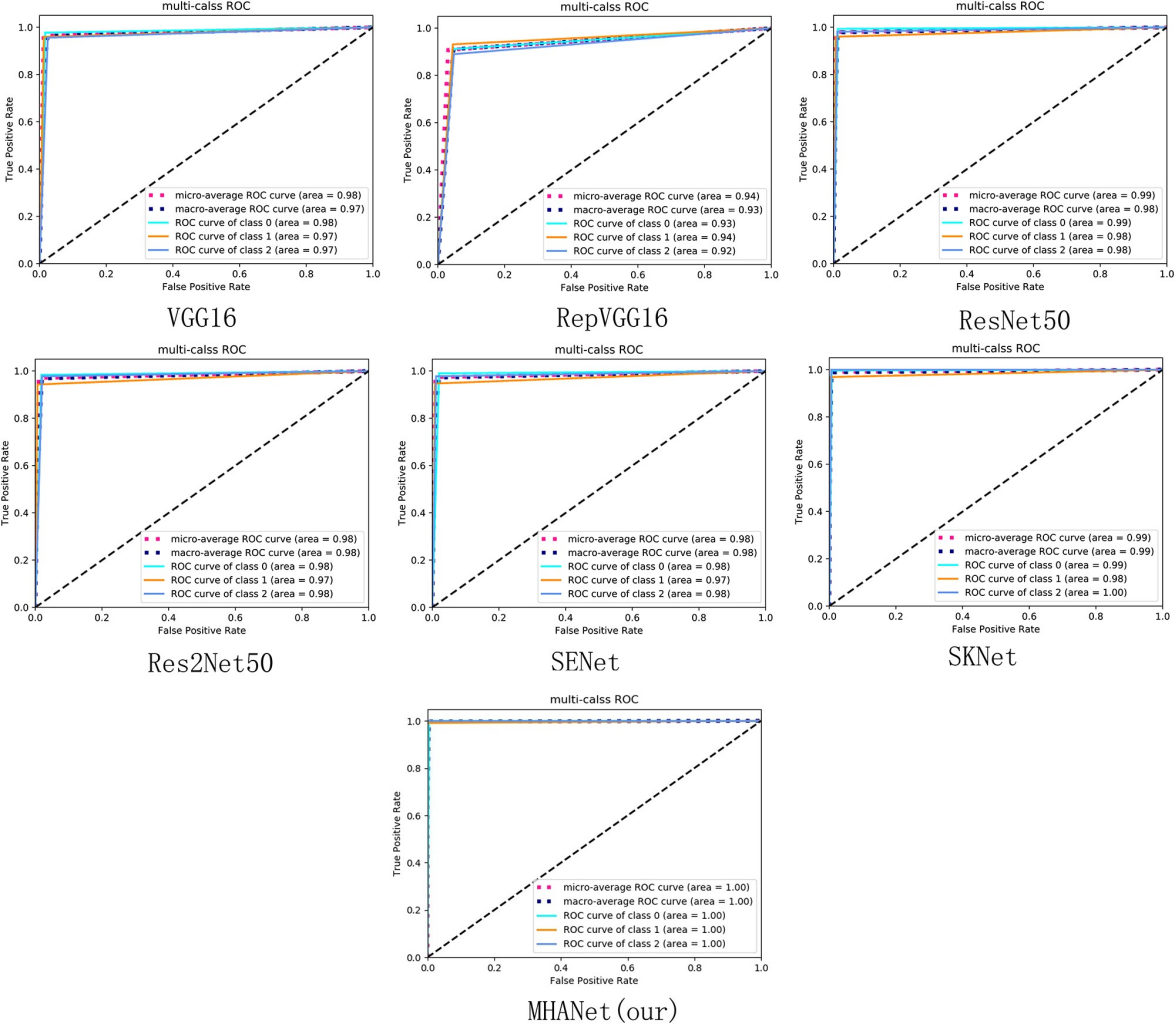
The micro-average ROC curve is obtained by the micro method in the sklearn.metrics.roc-auc-score function. The macro-average ROC curve is obtained by the macro method in the sklearn.metrics.roc-auc-score function. Class 0, class 1, and class 2 in the figure represent AMD, DME, and NORMAL, respectively.

As shown in Fig 9, The AUC values obtained by SENet, SKNet and MHANet are all above 0.96 on Dataset 1 and the average AUC values of three types are all above 0.97,which shows that attention mechanism model we trained on Dataset 1 can well balance the precision and recall indicators. Among them, the AUC values of the MHANet model proposed in this paper all reached 1, which also shows that the hybrid attention mechanism can have a better effect in retinal disease classification experiments than using only the channel attention mechanism.

**Fig 10:**
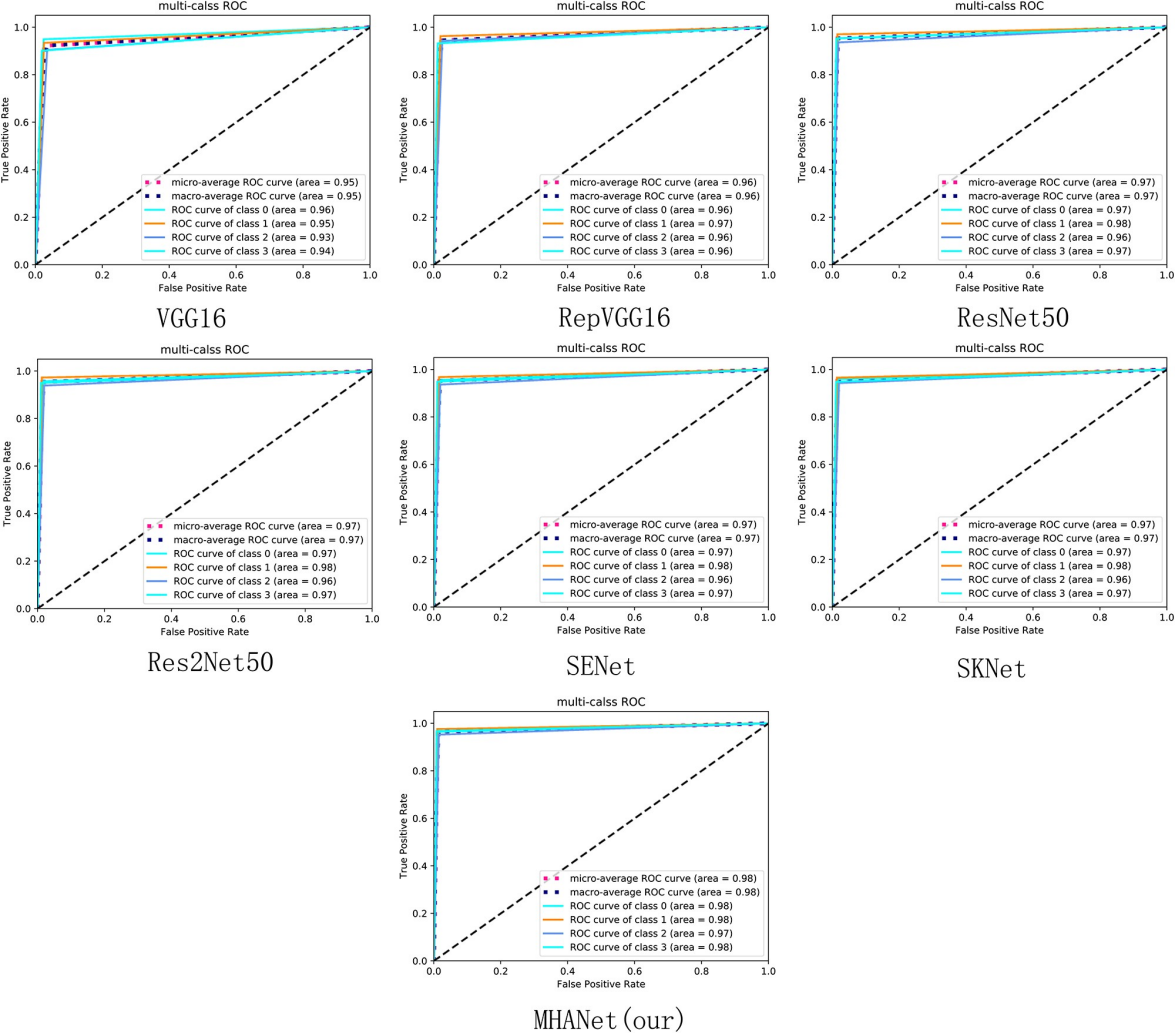
The micro-average ROC curve is obtained by the micro method in the sklearn.metrics.roc-auc-score function. The macro-average ROC curve is obtained by the macro method in the sklearn.metrics.roc-auc-score function. Class 0, class 1, and class 2 in the figure represent CNV, DURSEN, DME, and NORMAL, respectively.

As shown in Fig 10, The AUC values obtained by ResNet, Res2Net, SENet, and SKNet on Dataset 2 and the average AUC values of the three types are not much different, which shows that it is difficult to improve the discrimination ability of the network on Dataset2 using only the channel attention mechanism. The AUC value obtained on Dataset2 and the average AUC value of the three types of MHANet, which is composed of the parallel channel attention mechanism and the spatial attention mechanism, are the highest.

**Fig 11:**
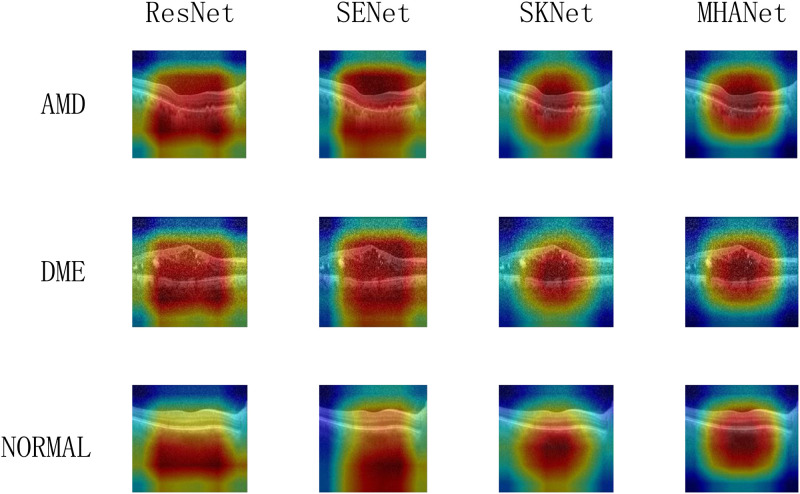
Heat map of Dataset1.

**Fig 12:**
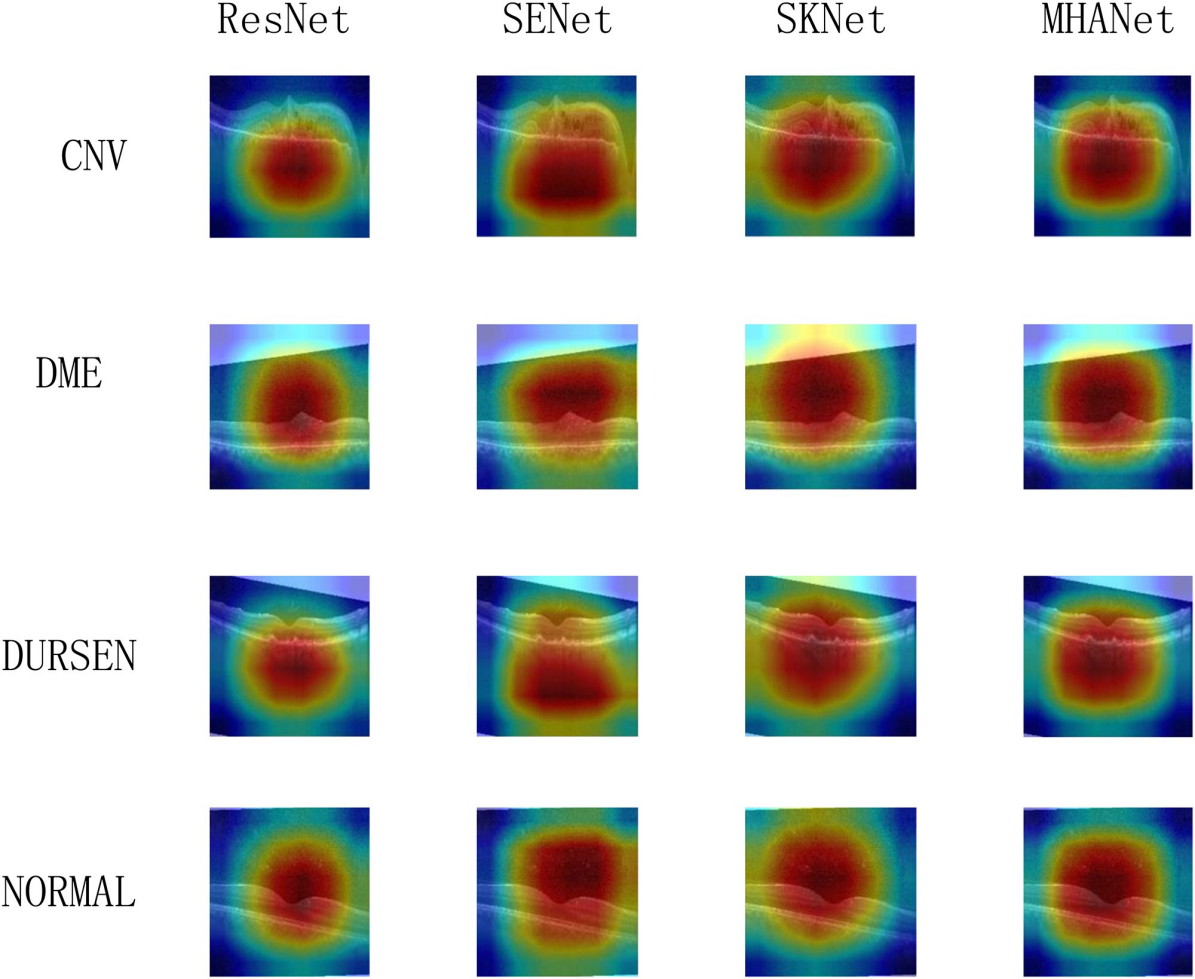
Heat map of Dataset2.

As shown in Figs 11 and 12, we show the focusing ability of different networks. Among them, ResNet50 without attention mechanism has completely different focusing ability on two different data sets. On the Dataset1, the focus range of ResNet50 is wide and the focus center does not accurately fall on the foreground information part. On the Dataset2, ResNet50 shrinks the focus range but the focus center still does not accurately fall on the foreground information part. SENet and SKNet adopt different channel attention mechanisms that cause their focusing abilities to be different. On the two datasets, the focusing ability of SKNet is obviously better than that of SENet. However, the focus centers of SENet and SKNet are still not accurately gathered in foreground information. The MHANet network we proposed has a small focus range on two different datasets and the focus center falls on the foreground information part.

## Conclusion

According to the distribution of lesion features in retinopathy images, we propose a hybrid attention mechanism to help the network lock foreground information in the image more accurately. The MHANet network module is composed of a parallel channel attention mechanism and a spatial attention mechanism. The channel attention mechanism assigns different channel coefficients to each channel feature map in the channel dimension, so as to highlight the channel feature map with the most abundant lesion features. The spatial attention mechanism assigns corresponding position coefficient to each element in the spatial dimension, so as to highlight the importance of elements in the lesion area. This hybrid attention mechanism can help the network focus on the lesion area in both the spatial dimension and the channel dimension. In this paper, the feasibility of the MHANet module is verified on two public retinopathy datasets. The experimental results show that the MHANet module can not only improve the accuracy of network classification and recognition, but also improve the generalization ability of the network.
